# Upconversion Nanoparticle-Based Dot-Blot Immunoassay for Quantitative Biomarker Detection

**DOI:** 10.1021/acs.analchem.4c00837

**Published:** 2024-06-13

**Authors:** Jakub Máčala, Ekaterina Makhneva, Antonín Hlaváček, Martin Kopecký, Hans H. Gorris, Petr Skládal, Zdeněk Farka

**Affiliations:** † Department of Biochemistry, Faculty of Science, Masaryk University, Kamenice 5, 625 00 Brno, Czech Republic; ‡ Institute of Analytical Chemistry of the Czech Academy of Sciences, Veveří 97, 602 00 Brno, Czech Republic

## Abstract

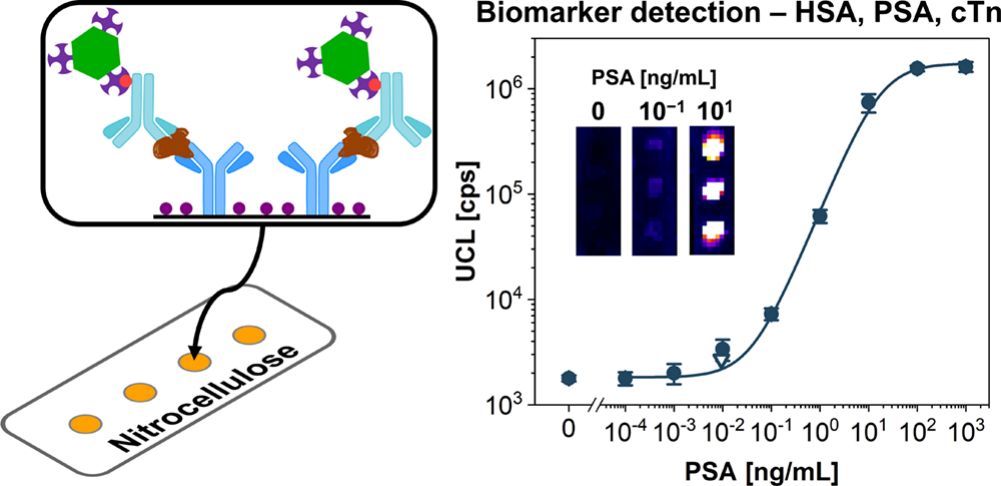

Dot-blot immunoassays are widely used for the user-friendly detection of clinical biomarkers. However, the majority of dot-blot assays have only limited sensitivity and are only used for qualitative or semiquantitative analysis. To overcome this limitation, we have employed labels based on photon-upconversion nanoparticles (UCNPs) that exhibit anti-Stokes luminescence and can be detected without optical background interference. First, the dot-blot immunoassay on a nitrocellulose membrane was optimized for the quantitative analysis of human serum albumin (HSA), resulting in a limit of detection (LOD) of 0.19 ng/mL and a signal-to-background ratio (*S*/*B*) of 722. Commercial quantum dots were used as a reference label, reaching the LOD of 4.32 ng/mL and the *S*/*B* of 3, clearly indicating the advantages of UCNPs. In addition, the potential of UCNP-based dot-blot for real sample analysis was confirmed by analyzing spiked urine samples, reaching the LOD of 0.24 ng/mL and recovery rates from 79 to 123%. Furthermore, we demonstrated the versatility and robustness of the assay by adapting it to the detection of two other clinically relevant biomarkers, prostate-specific antigen (PSA) and cardiac troponin (cTn), reaching the LODs in spiked serum of 9.4 pg/mL and 0.62 ng/mL for PSA and cTn, respectively. Finally, clinical samples of patients examined for prostate cancer were analyzed, achieving a strong correlation with the reference electrochemiluminescence immunoassay (recovery rates from 89 to 117%). The achieved results demonstrate that UCNPs are highly sensitive labels that enable the development of dot-blot immunoassays for quantitative analysis of low-abundance biomarkers.

## Introduction

Immunoassays represent a well-established family of bioanalytical tools that utilize antibodies for the detection and quantitation of various clinical and environmental analytes.^1,2^ The analysis is based on the formation of an immunocomplex between one or two antibodies and an antigen, with subsequent measurement of a signal generated by a label attached to one of the immunoreagents.^3^ Heterogeneous immunoassays are performed on a variety of solid phases, including microtiter plates (MTPs), membranes, magnetic microparticles, and polystyrene beads.^4^ This format allows washing away the unbound molecules after each assay step, providing a low assay background.^5^

Procedures utilizing a membrane surface as the solid phase are usually classified as paper-based assays.^6^ The most common material for membrane fabrication is nitrocellulose; other less frequent options include nylon and polyvinylidene fluoride.^7,8^ Compared with standard polystyrene MTPs, nitrocellulose membranes provide a highly porous 3D-structured surface with a high number of binding sites for the capture of biomolecules.^9−11^ In addition, nitration of the cellulose results in a high binding affinity for proteins.^12^ Nitrocellulose membranes are widely used in western blot analysis as transfer membranes for protein visualization,^10,11^ as the main component of point-of-care (PoC) rapid lateral flow immunoassay (LFIA) test strips,^9^ and in dot-blot immunoassays.

Dot-blot is a simple immunoassay technique in which proteins are directly deposited on the membrane surface and adsorbed through noncovalent interactions.^13^ This user-friendly method does not require expensive instrumentation and can be applied for rapid PoC analysis of analytes such as infectious agents, clinically relevant antibodies, and biomarkers.^14−17^ Many types of labels have been used for signal generation in dot-blots, mainly enzymes^18,19^ and fluorophores.^20^ However, these conventional labels typically have some drawbacks, e.g., the limited stability of enzymes or photobleaching in the case of fluorophores. In addition, the majority of dot-blot assays have rather limited sensitivity, leading to only qualitative or semiquantitative analysis.^21^ To address these disadvantages, alternative nanomaterial-based labels have been extensively studied with advantages such as improved stability and the possibility to fine-tune the properties by adjusting the nanomaterial composition or size.^1,22−24^ The typical examples of these nanomaterials are gold nanoparticles as colorimetric labels^14^ and quantum dots as an alternative to fluorescent dyes.^25^

Photon-upconversion nanoparticles (UCNPs) are another emerging class of nanomaterial-based labels.^26,27^ Under near-infrared excitation, these lanthanide-doped nanocrystals emit light of shorter wavelengths (anti-Stokes emission),^28^ eliminating background autofluorescence and improving the detection sensitivity.^29^ Furthermore, UCNPs are photostable even when exposed to high excitation powers.^30^ We previously employed UCNPs as a label in MTP-based upconversion-linked immunosorbent assay (ULISA), enabling the detection of clinically relevant biomarkers with high sensitivity.^31−34^ UCNPs were also employed in LFIA assays, for example, for the detection of SARS-CoV-2 nucleocapsid protein with a limit of detection (LOD) of 3.59 pg/mL^35^ or matrix metalloproteinases-8, interleukin-1 beta, and tumor necrosis factor alpha with LODs of 5.5, 0.054, and 4.4 ng/mL, respectively.^36^ However, only one study has used UCNPs as labels for the sensitive quantitation of analyte concentrations in dot-blot. Misiak et al.^37^ used dextran-coated UCNPs conjugated with protein G for the detection of various protein targets, such as murine monoclonal antibodies, obtaining the LOD of 0.19 μg/mL. However, this study was not able to achieve sub-ng/mL detection limits, which are necessary for the sensitive detection of many low-abundance clinical biomarkers (defined by Kim et al.^38^ as biomarkers with concentrations below 10 ng/mL).

Human serum albumin (HSA) is the most abundant protein in plasma. However, the concentration of HSA in the urine of a healthy population is in the range of 2.2–25 μg/mL, and elevated levels of HSA indicate kidney malfunction.^39^ Prostate-specific antigen (PSA) is the most important marker of prostate cancer.^40^ The concentration threshold, above which the patient should undergo further testing, is 3 ng/mL.^41^ Nevertheless, the detection of even lower PSA concentrations is important when radical prostatectomy is part of prostate cancer treatment. The PSA levels after surgery are typically near zero, and it is necessary to monitor even small changes in PSA concentration on the scale of picograms as an indication of cancer recurrence.^42^ Human cardiac troponin (cTn) is a biomarker of myocardial infarction.^43^ Healthy individuals have cTn blood concentrations of 1 to 50 pg/mL. However, damage to the heart tissue during myocardial infarction results in a significant release of cTn from the damaged cells, and cTn concentrations can increase to 50–100 ng/mL.^44^

**Figure 1:**
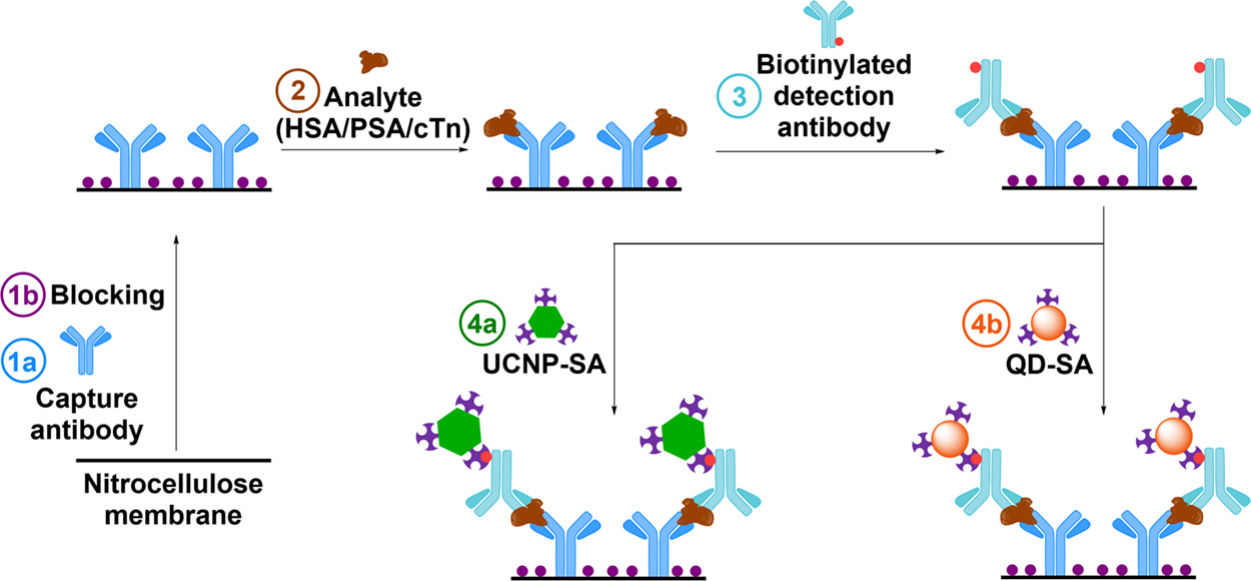
Scheme of a dot-blot immunoassay: (1a) coating with a capture antibody, (1b) blocking of the nitrocellulose membrane, (2) incubation with the analyte (HSA, PSA, or cTn), and (3) incubation with a biotinylated detection antibody. Different labels were used in the last immunoassay step: (4a) UCNP-SA for upconversion luminescence detection or (4b) QD-SA for fluorescence detection.

Here, we have developed a quantitative nitrocellulose-based dot-blot immunoassay utilizing UCNP labels (Figure 1). First, the immunoassay was optimized using HSA as an analyte. The performance of streptavidin-conjugated UCNPs (UCNP-SA) as labels was compared with the widely used streptavidin-conjugated quantum dots (QD-SA) as a reference. Additionally, HSA detection was carried out in real samples of spiked urine. Afterward, the dot-blot immunoassay was modified for the detection of other relevant clinical biomarkers, including PSA and cTn. To our knowledge, this is the first report of a dot-blot assay using the UCNP labels that achieves detection limits in the sub-ng/mL range.

## Experimental Section

### Chemicals and Materials

HSA, Tween-20, streptavidin-modified Cd-based core/shell quantum dots with an emission wavelength of 580 nm (QD-SA; OCNQSS580), anti-HSA polyclonal antibody (IgG fraction; A0433), fetal bovine serum, bovine serum albumin (BSA), and Protran nitrocellulose membranes (pore size of 0.1 and 0.45 μm) were purchased from Merck (Germany). PSA antigen (ab78528) and monoclonal anti-PSA antibody (ab403) were purchased from Abcam (UK). Anti-HSA monoclonal antibody (clone AL-01) was purchased from Exbio (Czech Republic). Biotinylated polyclonal anti-PSA antibody (BAF1344) was purchased from Bio-Techne (USA). SuperBlock TBS (SB) and Protein-Free blocking buffer TBS (PFB) were purchased from Thermo Fisher Scientific (USA). Cardiac troponin (cTn) I-T-C complex and monoclonal anti-cTnI-antibody clones 19C7cc, MF4c, and 560cc were purchased from Hytest (Finland). Other chemicals and reagents were purchased from Carl Roth (Germany), Penta (Czech Republic), or P-LAB (Czech Republic).

Clinical samples of human serum for PSA analysis were provided with written consent from all participants; the study was approved by the Ethics Committee of the University Hospital Brno (project number 24/22). The reference data on PSA concentrations were obtained by the Elecsys electrochemiluminescence immunoassay analyzer (Roche, Germany). Urine samples for HSA analysis were provided by two healthy volunteers and were purified using an Amicon Ultra centrifugal filter (molecular weight cutoff of 10 kDa; Merck, Germany) to remove potentially present HSA before the spiking.

Buffers included coating buffer (CB; 50 mM NaHCO_3_/Na_2_CO_3_, 0.05% NaN_3_; pH 9.6), washing buffer (WB; 50 mM Tris, 150 mM NaCl, 0.05% NaN_3_, 0.05% Tween-20; pH 7.5), and blocking buffers (BB; containing different amounts of blocking reagents diluted in washing buffer, or their mixtures v/v: 20, 50, or 100% PFB; 20, 50, or 100% SB, a mixture of 20% PFB and 20% SB; 1% w/v BSA). The assay buffers shared the same general composition (50 mM NaH_2_PO_4_/Na_2_HPO_4_, 150 mM NaCl, 1 mM KF, 0.1% PEG (*M*_W_ of 6000 Da), 0.02% Tween-20, 0.05% NaN_3_; pH 7.5), differing in the used blocking reagent: assay buffer 1 (AS1) contained 10% SB, assay buffer 2 (AS2) contained 10% SB and 10% PFB, and assay buffer 3 (AS3) contained 10% PFB.

Protocols for the synthesis of NaYF_4_:Yb^3+^,Er^3+^-based UCNPs and their conjugation with streptavidin via an alkyne-PEG-neridronate linker, characterization of the UCNPs and UCNP-SA conjugate using transmission electron microscopy (TEM) and dynamic light scattering (DLS), biotinylation of anti-HSA detection antibody, reference MTP-based ULISA assays, and dot-blot immunoassay based on QDs are provided in the Supporting Information.

### UCNP-Based Dot-Blot for the Detection of HSA

All steps were carried out at room temperature unless noted otherwise. A nitrocellulose membrane was cut into strips of 2.5 cm in length and 0.5 cm in width. Four 1 μL droplets of AL-01 monoclonal antibody in CB (50 to 250 μg/mL) were dispensed onto each strip, resulting in four measuring spots with a diameter of approximately 3 mm. The strips were allowed to dry for either 15 min, 60 min, or 18 h. Then, each strip was placed individually into a 2 mL microtube containing 1.9 mL of BB, and the tubes were gently agitated on a 3D shaker for 1 h. Afterward, the strips were put into a new set of microtubes containing serial dilutions of HSA (from 10^–3^ to 10^5^ ng/mL in assay buffer or 25% urine in assay buffer, 1.9 mL each). The strips were slowly agitated for either 15, 30, or 60 min on the 3D shaker. Then, the strips were transferred to washing trays containing 4 mL of WB each and placed on an orbital shaker. The strips were washed twice for 1 min, once for 10 min, and three times for 5 min under slow agitation; the WB was exchanged between the steps. After washing, the strips were placed into microtubes containing 1.9 mL of biotinylated anti-HSA polyclonal antibody solution (0.25 to 0.75 μg/mL). After incubating the strips for either 15, 30, or 60 min on the 3D shaker, the strips were washed using the previously described procedure. Subsequently, the strips were placed into another set of microtubes containing 1.9 mL of UCNP-SA conjugate dispersion (6.5 or 13 μg/mL). The strips were incubated for either 15, 30, or 60 min on the 3D shaker, followed by the same washing procedure as before with two additional 5 min washing steps. Finally, the strips were dried for 30 min at 40 °C and scanned.

### UCNP-Based Dot-Blot for the Detection of PSA and cTn

The optimized immunoassay was subsequently used for the detection of PSA and cTn. The PSA detection was based on the coating antibody ab403 (100 μg/mL) and the biotinylated detection antibody BAF1344 (0.25 μg/mL), analyzing serial PSA dilutions from 10^–4^ to 10^3^ ng/mL in assay buffer or 50% fetal bovine serum in assay buffer. For the clinical sample analysis, the serum was diluted 10× in 50% fetal bovine serum in assay buffer. In the case of cTn, a 1:1 mixture of MF4c and 19C7cc antibodies (final concentration of each antibody in the mixture of 100 μg/mL) was used for the coating, and biotinylated antibody 560cc (0.5 μg/mL) was used as the detection antibody; the biotinylation was done according to the previously published protocol.^31^ Serial dilutions of the cTn antigen from 10^–3^ to 10^4^ ng/mL were analyzed in assay buffer or 25% fetal bovine serum in assay buffer. The serum dilutions for PSA and cTn assays were based on our previous experiments.^32,34^

### Data Acquisition and Evaluation

The upconversion luminescence was measured by an UPCON S-Pro reader (Labrox, Finland). A 980 nm laser with a 976/30 nm excitation filter was used for the excitation of UCNPs, and the emission was collected through a 540/60 nm emission filter utilizing a D800 dichroic mirror (950–1000 nm excitation, 500–720 nm emission). The strips were scanned using a step size of 0.5 mm and a signal integration time of 500 ms for each point. The data were imported to ImageJ software (National Institutes of Health, USA), scan images were constructed utilizing pseudocolor scale, and average intensities of the detection spot areas were calculated. The following data evaluation was done using OriginPro 2023 software (OriginLab, USA). For each analyte concentration, the mean and standard deviation were calculated from the intensity values of the four detection spot replicates on the strip. The intensity values were fitted using a logistic function:
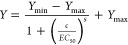
where *Y* represents upconversion luminescence (or fluorescence in the case of QD-SA labels), *Y*_min_ is the minimum of the sigmoidal curve, which corresponds to the assay background, *Y*_max_ is the maximum of the sigmoidal curve, *c* is the analyte concentration, *EC*_50_ is the half-maximal effective concentration, and *s* is the slope at the inflection point. The LODs were estimated from the regression curves as the concentrations corresponding to the *Y*_LOD_ value:
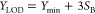
where *Y*_min_ is the background value obtained by the logistic fit and *S*_B_ represents the standard deviation of the blank.^32^ The signal-to-background (*S*/*B*) ratios were calculated using the intensities obtained for the analyte concentration of 10^3^ ng/mL and the corresponding blank (0 ng/mL). The standard deviations of the *S*/*B* values were calculated considering the propagation of uncertainties.

## Results and Discussion

### Optimization of UCNP-Based Dot-Blot for HSA

In dot-blot immunoassays, the analyte is commonly detected by a colorimetric change, which typically leads to qualitative or semiquantitative results.^45^ By contrast, our goal was to utilize UCNPs (characterization by TEM and DLS provided in Figure S1) as highly sensitive labels to convert dot-blots into a reliable and quantitative method. By adjusting each of the immunoassay parameters and carefully studying their influence on the assay performance, we found the optimum conditions for the UCNP-based dot-blot.

**Figure 2:**
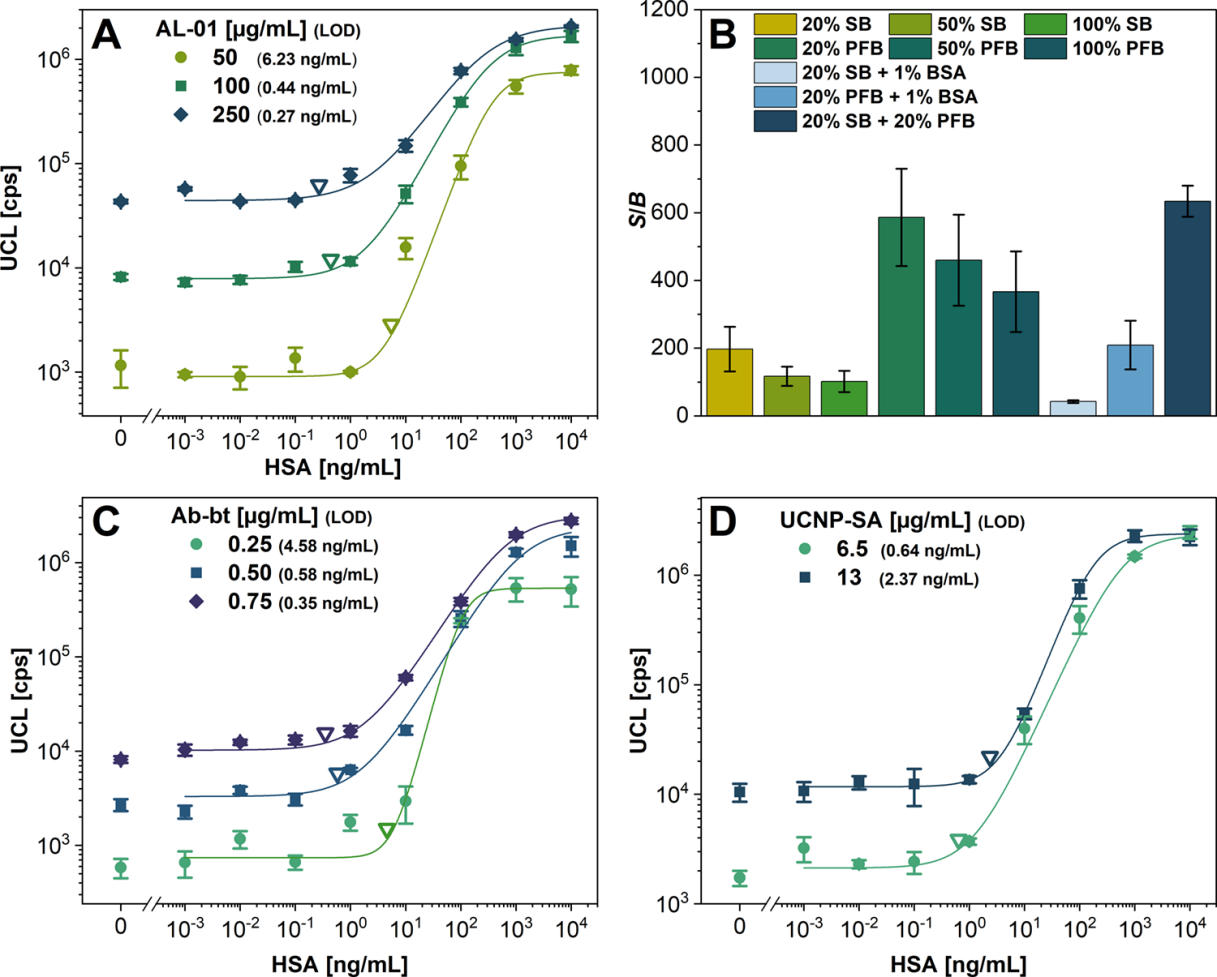
Optimization of UCNP-based dot-blot for HSA. (A) Calibration curves using different coating antibody concentrations. (B) Dependence of the *S*/*B* ratio on various blocking solutions. Calibration curves for different concentrations of the (C) biotinylated detection antibody and (D) UCNP-SA label. Error bars represent standard deviations, and empty triangles indicate the LODs.

A nitrocellulose membrane with a pore size of 0.45 μm was used for all the optimization experiments. First, the concentration and the incubation time of the AL-01 coating antibody were optimized (Figure 2A). As the antibody concentration increased, a decrease of *S*/*B* was observed (Figure S2A), connected with the increasing background signals. However, increasing the coating antibody concentration still resulted in a better LOD. Therefore, an AL-01 concentration of 100 μg/mL was chosen as a compromise between the high *S*/*B* (157) and low LOD (0.44 ng/mL). Additionally, three coating times of 15 min, 60 min, and 18 h were tested (Figure S2B). The coating time of 15 min resulted in the best *S*/*B* of 296; the longer incubation periods led to decreased *S*/*B* values, probably due to the capture antibody denaturation during the excessive drying.

The next step was the optimization of the blocking conditions (Figures 2B and S3). It was particularly important to minimize the nonspecific binding of proteins on the porous membrane structure, as nonspecific binding can result in a high assay background and insufficient LODs. Different concentrations of SuperBlock (SB) buffer containing purified glycoproteins or Protein-Free blocking buffer (PFB) containing nonprotein compounds were tested. Additionally, their mixture and subsequent blocking with BB and BSA solution were also investigated. The data indicated that the mixtures of the components enabled blocking the nitrocellulose membrane more efficiently than individual blocking agents. The most efficient blocking was obtained for 1 h incubation using the mixture of 20% SB and 20% PFB in WB, reaching the *S*/*B* ratio of 672.

Afterward, the biotinylated detection antibody concentration was optimized (Figures 2C and S2C). With the increasing detection antibody concentration, the signals for higher HSA concentrations increased. However, as the assay background also increased, a decreasing trend of *S*/*B* was observed. Nevertheless, the increased detection antibody concentrations led to better LODs. The detection antibody concentration of 0.5 μg/mL was chosen as optimal because it provided the best compromise between sufficiently high *S*/*B* (478) and low LOD (0.58 ng/mL).

Optimizing the UCNP-SA concentration was crucial to avoid increased background levels due to nonspecific binding of the labels to the membrane surface. On the other hand, a low label concentration would lead to low signal intensities and, therefore, a reduced sensitivity. Two UCNP-SA concentrations of 6.5 and 13 μg/mL were tested (Figures 2D and S2D). The slope of the calibration curve for both concentrations was similar; however, there was a significant difference in the background intensities caused by the increased nonspecific binding in the case of the higher UCNP-SA concentration. For the lower concentration, the background level was 6.1 times lower, resulting in an *S*/*B* ratio of 858. The LOD obtained with the lower label concentration was 0.64 ng/mL, 3.7 times better than with the higher one. Hence, the lower label concentration was chosen as optimal, as it benefited from the lower background as well as better LOD.

Next, the incubation time of the strips with the analyte, biotinylated detection antibody, and UCNP-SA was optimized. It was necessary to find an adequate time for sufficient binding of assay components while considering that too long incubation could lead to more pronounced nonspecific binding, as it provides more time for nonspecific interactions between assay components and adsorption of labels to the nitrocellulose membrane. With the increasing incubation time, a significant increase in intensities was observed for both the blanks and the high HSA concentrations. However, the background increase was more pronounced, strongly deteriorating the *S*/*B* ratio and LOD (Figure S4A,B). Thus, the incubation time of 15 min was chosen as the most suitable, as it resulted in the best assay parameters as well as a shorter assay length.

Three different assay buffer compositions were tested, all based on phosphate buffer but with varying blocking reagents (Figure S4C,D). The blocking reagent is typically added to the assay buffer to prevent nonspecific binding, as the previously attached blocking molecules can be partially washed away during the incubation steps and to reduce nonspecific interactions between the biomolecules. The LODs were similar for all the tested options; AS3 containing 10% PFB resulted in the best *S*/*B* ratio of 801. Thus, AS3 was chosen as the most suitable assay buffer for the subsequent experiments.

Finally, nitrocellulose membranes with different pore sizes (0.1 and 0.45 μm) were compared (Figure S4E,F). The decreasing pore size generally provides a higher surface area for immobilizing the capture antibody; however, it also provides more space for nonspecific binding. The signal intensity obtained for the HSA concentration of 10^3^ ng/mL was 2.9 times higher utilizing the membrane with 0.1 μm pore size; however, the background increased 5 times. This shows that the higher surface area increased the nonspecific binding more than the specific signals. The resulting *S*/*B* values were 372 and 722, and LODs of 0.36 and 0.19 ng/mL for membranes with the pore size of 0.1 and 0.45 μm, respectively. Therefore, the membrane with a pore size of 0.45 μm was selected for further experiments.

### Performance of UCNP-Based Dot-Blot for HSA and Comparison with QDs

**Figure 3:**
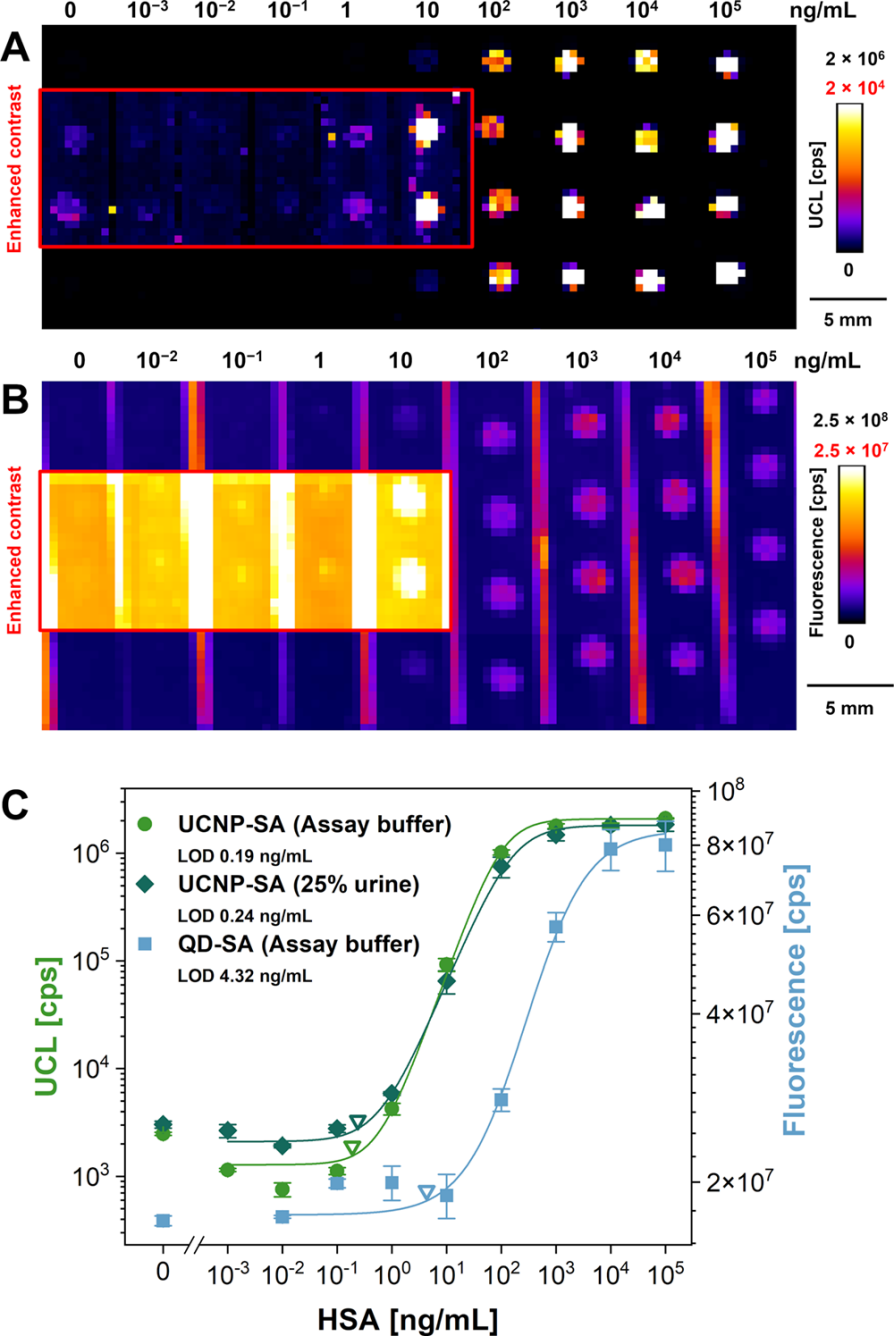
Intensity scans in pseudocolor scale of the UCNP-based dot-blot for the detection of HSA in buffer with (A) UCNP-SA detection labels and (B) QD-SA detection labels. The areas marked with red rectangles show the data with enhanced contrast (red cutoff values in the intensity scales). (C) Calibration curves for the detection of HSA using UCNP-SA labels in the buffer and in the 25% urine and QD-SA labels in the buffer. The HSA concentrations are indicated above the scans. The intensity scales in panels A and B were chosen to facilitate the visibility of lower signals; the graph in panel C was evaluated based on raw signal values. Error bars represent standard deviations, and empty triangles indicate the LODs.

By utilizing the optimized assay parameters (as summarized in Table S1), the HSA detection in buffer reached the *S*/*B* ratio of 722 and the LOD of 0.19 ng/mL (Figure 3A,C). Aitekenov et al.^46^ published a review summarizing methods for detecting and quantifying proteins in urine, including immunoassays. The LOD for HSA obtained by our dot-blot assay was 1 to 2 orders of magnitude lower than in most of the other reported assays, demonstrating the outstanding performance of the UCNP-based dot-blot immunoassay.

QD-SA labels were tested to compare the performance of UCNPs with other types of luminescent nanoparticles. Two QD-SA conjugate concentrations of 1 nM (Figure S5) and 5 nM were investigated (Figure 3B,C). The background intensities reached similar values; however, for higher HSA concentrations, the signals were significantly higher with 5 nM QD-SA. Nevertheless, the LOD of 4.32 ng/mL and the *S*/*B* ratio of 3.4 were not even close to the performance of UCNP-based assay (23-fold difference in LOD and 212-fold difference in the *S*/*B* ratio), which can be explained by the background-free anti-Stokes emission of UCNPs under 980 nm excitation. Under these conditions, the nitrocellulose membrane did not show any autofluorescence, unlike under 340 nm excitation light for the excitation of QD labels (Table S2). Compared with polystyrene MTP, the autofluorescence of nitrocellulose was 8 times higher utilizing the optical setup used for the QD detection. In contrast, the setup for UCNPs showed only minor variation between the MTP and the nitrocellulose (1.2-fold difference).

Additionally, the photostability of QDs and UCNPs was examined, as QDs are widely recognized for their high photostability compared with conventional organic fluorophores.^47^ A nitrocellulose strip with an HSA concentration of 100 ng/mL labeled with either QD-SA or UCNP-SA conjugate was measured over time, with both strips being stored under the same conditions exposed to daylight (Figure S6). The fluorescence intensity of QDs decreased by 28% after 15 days of storage, while the upconversion luminescence of UCNPs decreased by only about 1.5% during the same interval, demonstrating the higher photostability of UCNPs. These results highlight the advantages of UCNPs over other types of luminescent nanoparticles, such as QDs.

### UCNP-Based Dot-Blot Immunoassay for the Detection of HSA in Real Samples

To determine the performance of the UCNP-based dot-blot for the real sample analysis, spiked samples of urine were analyzed. Real samples often negatively affect the assay performance, mainly due to the increased level of nonspecific interactions caused by the complex sample matrix. Hence, the assay was conducted with a sample containing 25% urine in the assay buffer, as the dilution reduces the influence of the sample matrix on the assay performance. Moreover, the blocking agent present in the assay buffer reduces nonspecific interactions. Consequently, when real samples from different patients are analyzed, the dilution helps diminish the differences between the respective sample matrices. The real sample assay achieved an *S*/*B* ratio of 490 and LOD of 0.24 ng/mL (calibration curve shown in Figure 3C, with intensity scan in Figure S7A). The *S*/*B* decrease compared with the assay in buffer was caused by the slightly increased background; however, the LOD was affected only marginally, and the overall assay performance was not significantly influenced. Additionally, urine from two healthy volunteers was spiked with HSA concentrations ranging from 3 to 300 ng/mL and analyzed by the UCNP-based dot-blot (Figure S7B). The data obtained by the dot-blot detection corresponded well with the spiked concentrations for both tested samples (recovery rates from 79 to 123%; *R*^2^ of 0.98 and 0.97), demonstrating the potential of UCNP-based dot-blot for the clinical analysis of HSA levels in urine.

### UCNP-Based Dot-Blot for the Detection of PSA and cTn

**Figure 4:**
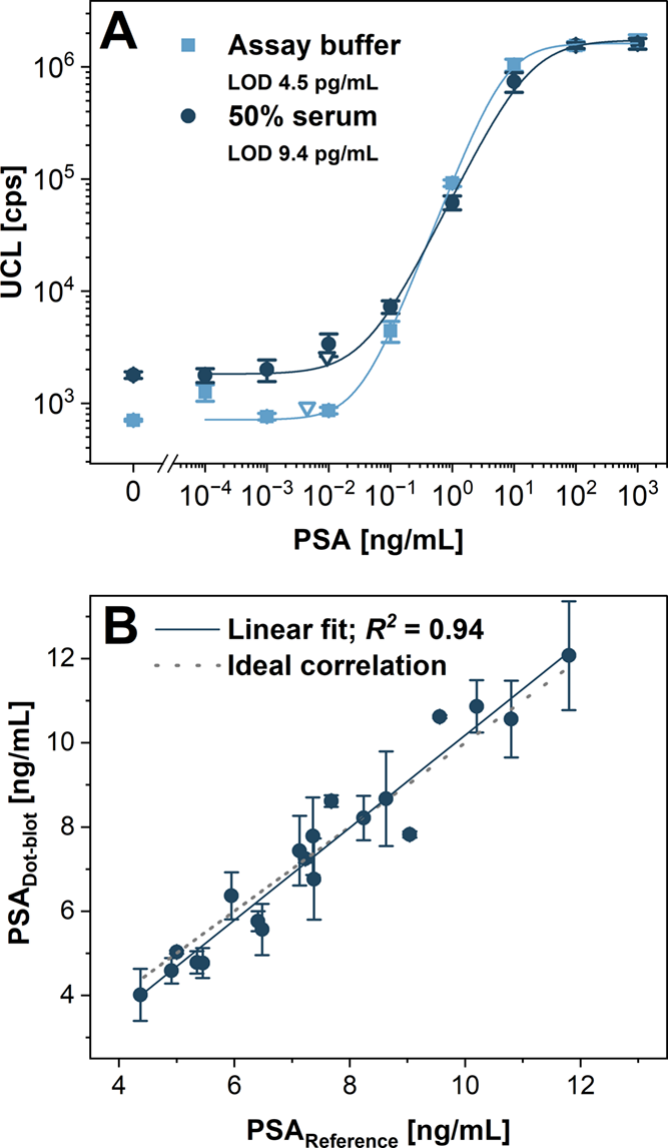
(A) Calibration curves of the UCNP-based dot-blot detection of PSA in assay buffer and 50% serum. (B) Correlation between PSA concentrations in the clinical samples found by the dot-blot detection and by reference electrochemiluminescence immunoassay. Error bars represent standard deviations, and empty triangles indicate the LODs.

To prove the versatility and robustness of the developed dot-blot immunoassay, the determination of PSA as a prostate cancer biomarker and cTn as a biomarker of myocardial infarction was performed under the optimized assay conditions. The assays were carried out in the buffer and spiked fetal bovine serum to mimic the complex blood matrix, the typical sample used for the clinical analysis of PSA and cTn.^32,34^ Initially, the detection of PSA was performed in the AS3 buffer. An *S*/*B* ratio of 2397 and LOD of 4.5 pg/mL proved the sensitive detection of this biomarker. The detection in 50% serum in AS3 resulted in an *S*/*B* ratio of 906 and an LOD of 9.4 pg/mL (Figures 4A and S8). The *S*/*B* ratio decreased for the detection in serum because of the more nonspecifically bound labels, resulting in increased background levels. Consequently, the LOD increased approximately two times due to the background increase; however, it was still sufficient to allow the detection of low PSA levels present after the radical prostatectomy.^42^

To further confirm the applicability of the UCNP-based dot-blot for prostate cancer diagnostics, clinical samples of human serum were analyzed, and the found PSA levels were compared with concentrations provided by a standard electrochemiluminescence immunoassay (Figure 4B). The dot-blot results were in strong agreement with the results obtained by the reference method (recovery rates from 89 to 117%; *R*^2^ of 0.94), showing the potential of UCNP-based dot-blot for the clinical sample analysis. Moreover, the unique optical properties of UCNPs make them suitable for the detection with low assay background, consequently reaching low LOD values.

A similar procedure was carried out for cTn (Figure S9). The detection in buffer yielded an *S*/*B* ratio of 399 and LOD of 0.29 ng/mL; the assay in 25% serum resulted in an *S*/*B* ratio of 378 and LOD of 0.62 ng/mL. In the case of cTn, the blank signals were slightly lower for the detection in the real sample compared with the AS3 buffer. This is probably a result of the adsorption of serum proteins to the membrane surface, resulting in additional blocking of the binding sites. Nevertheless, there was a decrease in the *S*/*B* ratio in the real sample analysis. This was caused by the lower signal intensities of the high analyte concentrations, as serum proteins can also block some of the binding sites of the capture antibodies. In addition, the relative intensity increase between the samples with 0.1 and 1 ng/mL of cTn was more pronounced in the assay buffer, resulting in an earlier rise in the calibration curve. Due to this, the LOD in 25% serum increased approximately twice compared with the detection in buffer. Nevertheless, it still enables the detection of elevated cTn levels found in connection with myocardial infarction.^44^ Overall, the complex sample matrix had a similar effect on the LOD for PSA and cTn.

### Comparison of UCNP-Based Dot-Blot Performance with ULISA in MTP

To further assess the performance of the UCNP-based dot-blot, we compared the results with MTP-based assays (Table 1 and Figure S10), which were carried out according to our previously published protocols.^32,33,48^ The achieved LODs in the real samples were 125 pg/mL of HSA in 25% urine, 2.5 pg/mL of PSA in 50% serum, and 39 pg/mL of cTn in 25% serum. In all cases, the dot-blot assays resulted in LODs that were 1 order of magnitude higher as compared with MTP assays. The difference was mainly caused by stronger nonspecific binding in the membrane-based assays, which originated in the nature of the membrane material itself. The porous membrane surface provides more binding sites for the biomolecules than the polystyrene MTP well. This enabled reaching higher signal intensities in the membrane-based assays; for instance, the signal obtained for 100 ng/mL of PSA in the membrane assay was 5.4× higher compared with the MTP assay. However, the large number of binding sites also increases the potential for nonspecific binding, resulting in elevated background levels. The *S*/*B* ratios of the dot-blot assays for the detection of HSA and cTn in the buffer were lower compared with the MTP assays due to the higher background of the dot-blots. However, for PSA, whose dot-blot detection was the most sensitive among the tested analytes, the *S*/*B* ratio (for 100 ng/mL of PSA) was 7.6 times higher than in the MTP assay. Overall, a similar trend of variation of LODs for the different analytes was visible in both the dot-blot and the ULISA detection approaches. The origin of this variation lies in the use of different immunoreagents, as the immunoassay sensitivity generally does not depend only on the label but also on the affinity of the used antibodies toward the detected antigen.^49^

**Table 1:** Summary of the Analytical Parameters of UCNP-Based Dot-Blot Assay and ULISA in MTP for HSA, PSA, and cTn

analyte	sample matrix	UCNP-based dot-blot		ULISA in MTP	
		LOD [pg/mL]	*S*/*B*	LOD [pg/mL]	*S*/*B*
HSA	buffer	190	722	25	867
	25% urine	240	490	125	277
PSA	buffer	4.5	2397	1.2	291
	50% serum	9.4	906	2.5	142
cTn	buffer	290	399	17	1760
	25% serum	620	378	39	219

In the real sample analysis, the *S*/*B* ratios for all three analytes were higher in dot-blot assays than with the MTPs, however, for different reasons. In the case of HSA and PSA detection, a signal decrease for high sample concentrations was observed in both the MTP and the dot-blot assays, caused by the blocking of the surface binding sites by the complex matrix. This effect was more pronounced in MTP-based assays, due to the lower binding capacity of the polystyrene MTP compared with the nitrocellulose membrane. On the other hand, the detection of cTn in real samples showed a more pronounced increase of the background signal in MTP than with the dot-blot, connected with nonspecific adsorption of serum components and cross-reactivity of the detection antibody. Together, these observations highlight the advantages of dot-blot assay compared with the MTPs in real sample analysis.

Compared with the MTP assay, the dot-blot assay needed less time for the preparation and performance of the assay (4 h from the start of the coating to the scanning) because there was no need for overnight coating of the detection surface and each incubation step only took 15 min instead of 1 h for the ULISA. The UCNP-based dot-blot meets the requirements for the practical detection of all three analytes in their respective real samples,^42,44,50^ confirming the suitability of the assay for clinical analysis.

### Comparison of UCNPs with Other Labels for Dot-Blot-Based Biomarker Assays

Only a few articles describe the detection of the same analytes using the dot-blot technique. Matsuda et al.^51^ published a semiquantitative immunoassay for the detection of proteins in urine based on colloidal silver staining. This assay lacked selectivity and detected all the proteins in urine with the LOD of 2.5 μg/mL. By contrast, our dot-blot assay was quantitative and selective toward HSA (with the possibility of adjusting the selectivity by changing the antibodies), and the achieved LOD in the urine sample was 4 orders of magnitude lower. Khramtsov et al.^52^ reported on a dot-blot immunoassay for PSA based on solid-phase NMR. They conjugated carbon-encapsulated iron nanoparticles with anti-PSA antibodies and used them as magnetic labels. The dot-blot assay was carried out within 4 h and achieved an LOD of 0.44 ng/mL in 50% serum. In contrast, our assay achieved 47-fold lower LOD while maintaining a similar total assay duration. Finally, Dorraj et al.^53^ and Guo et al.^54^ utilized gold nanoparticles with silver enhancement for semiquantitative dot-blot detection of cTn, obtaining the LOD of 5 ng/mL in plasma and 1 ng/mL in serum, respectively. Our dot-blot procedure for cTn provided an LOD of 0.62 ng/mL in real sample, achieving a lower LOD than in both earlier reports.

In addition, Misiak et al.^37^ reported on the use of UCNP labels in dot-blot immunoassays, however, focusing on different analytes. In their work, UCNPs were successively coated with a polyvinylpyrrolidone/vinyl alcohol copolymer, aminated dextran, and protein G. The prepared labels were then employed in semiquantitative detection of various targets, in particular, lipopolysaccharide from *Escherichia coli* (LOD of 1.95 μg/mL), murine monoclonal antibodies (LOD of 0.19 μg/mL), and immunoglobulin from human serum (LOD of 0.49 μg/mL). When comparing the LOD of murine antibodies that achieved the best performance, the LOD reported in our work is 1,000× , 42,000×, and 655× better for HSA, PSA, and cTn, respectively. This demonstrates that it is not only the nanoparticle core by itself but also its surface coating preventing nonspecific binding and optimized detection procedure, which are essential to achieve the lowest LOD possible.

Overall, our results surpassed the sensitivity of other dot-blot assays for the detection of HSA, PSA, and cTn previously reported in the literature. According to a review by Surti et al.,^45^ who discussed the recent progress in dot-blot immunoassays, the LODs reported in the literature range from μg/mL to pg/mL. Our results clearly demonstrate the benefits of using UCNPs as labels for highly sensitive and quantitative dot-blot analysis surpassing the performance of most published dot-blot assays.

## Conclusions

This study advances dot-blot assays for the quantitative detection of biomarkers by using UCNPs as highly sensitive optical labels. First, each assay step was thoroughly optimized using HSA as an analyte. Compared with QDs as a reference label, the UCNPs demonstrated outstanding signal intensities and low assay background, resulting in 2 orders of magnitude higher *S*/*B* ratio and 1 order of magnitude better LOD while maintaining a simple protocol and short assay time. The detection of HSA in real samples of urine demonstrated the robustness of the assay, reaching an LOD of 0.24 ng/mL and recovery rates from 79 to 123%. The versatility of the UCNP-based dot-blot was confirmed by adapting it to detect PSA and cTn. The obtained LODs in real samples were 9.4 pg/mL for PSA and 0.62 ng/mL for cTn, proving the ability of sensitive detection in complex matrices and making the developed assay suitable for clinical analysis. Furthermore, clinical samples of patients examined for prostate cancer were analyzed, achieving an excellent correlation with the reference electrochemiluminescence immunoassay (recovery rates from 89 to 117%). Compared with other dot-blot assays utilizing UCNP labels, the LOD of our method was 655 to 42,000-fold lower, depending on the analyte. The reported results thus represent a promising foundation for further progress of membrane-based immunoassays and microarrays.

## Supporting Information Available

Protocols for the synthesis and characterization of UCNP-SA labels, biotinylation of anti-HSA antibody, QD-based dot-blot immunoassay for HSA, ULISA assays for HSA, PSA, and cTn, and results from the optimizations of UCNP-based dot-blot immunoassays, dot-blot immunoassays with QD-SA labels, and ULISA assays (PDF)
