# Supplementary material for: Upconversion Nanoparticle-Based Dot-Blot Immunoassay for Quantitative Biomarker Detection
Source: Anal Chem. 2024 Jun 13;96(25):10237–45. doi: 10.1021/acs.analchem.4c00837 (PMC11209662; doi:10.1021/acs.analchem.4c00837)
Supplement: Supplementary file 1 — ac4c00837_si_001.pdf [file ac4c00837_si_001.pdf]

SUPPORTING INFORMATION
Upconversion nanoparticle-based dot-blot immunoassay for quantitative biomarker detection

Jakub Máčala,[a] Ekaterina Makhneva,[a] Antonín Hlaváček,[b] Martin Kopecký,[a] Hans H. Gorris,[a] Petr Skládal,[a] Zdeněk Farka[a,*]

[a] Department of Biochemistry, Faculty of Science, Masaryk University, Kamenice 5, 625 00 Brno, Czech Republic

[b] Institute of Analytical Chemistry of the Czech Academy of Sciences, Veveří 97, 602 00 Brno, Czech Republic

* Corresponding author. E-mail: farka@mail.muni.cz

Table of contents

1 Materials and methods S-3

1.1 Preparation of UCNP-SA labels S-3

1.2 Characterization of UCNPs S-4

1.3 Biotinylation of polyclonal anti-HSA antibody S-4

1.4 QD-based dot-blot immunoassay for HSA S-4

1.5 Upconversion-linked immunosorbent assays S-5

2 Results and discussion S-6

Figure S1: Characterization of UCNPs S-6

Figure S2: Optimizations of UCNP-based dot-blot for HSA S-7

Figure S3: Intensities of HSA samples and blanks obtained for different blocking S-8

Figure S4: Optimizations of incubation time, assay buffer, and membrane pore size S-9

Table S1: Summary of the optimized UCNP-based dot-blot assay parameters S-10

Figure S5: Dot-blot immunoassay for HSA detection with QD-SA labels. S-11

Table S2: Autofluorescence background intensities of the MTP and nitrocellulose S-11

Figure S6: Comparison of the photostability of QD-SA and UCNP-SA labels S-12

Figure S7: Detection of HSA in real samples S-12

Figure S8: Intensity scans of the UCNP-based dot-blot for the detection of PSA S-13

Figure S9: Intensity scans of the UCNP-based dot-blot for the detection of cTn S-13

Figure S10: Calibration curves of MTP-based ULISA S-14

3 References S-15

1 Materials and methods

1.1 Preparation of UCNP-SA labels

UCNPs with a composition of $NaYF_4$:Yb^{3+},Er^{3+} were synthesized by high-temperature co-precipitation according to our previously published protocol.[1,2]

To prepare the alkyne-PEG-neridronate linker, 30 mg of neridronate (Merck, Germany) were dissolved in 1 M aqueous solution of NaOH (128 µL). Then, 398 µL of phosphate buffer (PB; 50 mM NaH_2PO_4/Na_2HPO_4; pH 7.6) were added, and the mixture was sonicated for 5 min. Afterward, 500 µL of 15 mM solution of α-*N*-hydroxysuccinimide-ω-alkyne polyethylene glycol (M_W 3000 Da; Rapp Polymere, Germany) in PB were added and incubated with shaking for 2 h at room temperature (RT) followed by overnight incubation at 4 °C. The reaction mixture was then purified by dialysis against distilled water for 5 days (water was exchanged 3 times a day) using the Spectra/Por Float-A-Lyzer dialysis tube (500–1000 Da MWCO; Carl Roth, Germany). The purified linker solution was then transferred into a glass vial, lyophilized (Alpha 1-2, Christ, Germany) for 24 h, and stored at 4 °C.

For the conjugation of UCNPs with the alkyne-PEG-neridronate linker, 20 mg of UCNPs dispersed in cyclohexane (500 µL) were mixed with an equal volume of 200 mM HCl, incubated with shaking at 38 °C for 40 min, and sonicated for 30 min to exchange the oleic acid ligands for Cl^{-} ions, allowing a phase transfer from cyclohexane to water. The upper water phase was transferred to another tube, acetone was added in excess, and the mixture was centrifuged for 1 h at 1,000 *g*. Then, the solution was removed, and the UCNP pellet at the bottom of the tube was dispersed in 500 µL of distilled water, followed by 5 min of sonication. Alkyne-PEG-neridronate linker (3 mg) was dissolved in 500 µL of distilled water and added to the dispersion of UCNPs. The incubation was done overnight at 38 °C with constant shaking. The prepared conjugates were purified by dialysis against distilled water containing 1 mM KF for 3 days (solution exchanged 3 times a day) utilizing the Float-A-Lyzer dialysis tube (50 kDa MWCO).

Streptavidin (Thermo Fisher Scientific, USA) was modified using NHS-dPEG$_8$-azide linker (Merck, Germany). First, 31.25 µL of 200 mM linker solution in DMF were added to 150 µL of streptavidin solution (4 mg/mL) in PB, followed by the addition of 187.5 µL of PB. The reaction mixture was incubated with shaking for 2 h at RT, followed by terminating the reaction by the addition of 50 µL of WB. The prepared streptavidin-azide conjugate was purified by centrifuge filtration using an Amicon Ultra centrifugation filter (10 kDa MWCO; Merck, Germany) and stored at 4 °C in PB (final concentration of 1 mg/mL).

For the conjugation of UCNPs with streptavidin-azide, 100 µL of Tris buffer (375 mM; pH 7.5), 10 µL of 25 mM $CuSO_4$, and 10 µL of Tris(3-hydroxypropyltriazolylmethyl)amine (Merck, Germany) were mixed and added to the 10 mg of alkyne-PEG-neridronate-UCNPs (in 1.4 mL of distilled water containing 1 mM KF). The mixture was purged with argon for 40 min to remove any oxygen, followed by the addition of 100 µL of streptavidin-azide (1 mg/mL in PB) and purging for another 10 min. The click reaction was started by the addition of 10 µL of

20 mg/mL sodium ascorbate (Merck, Germany) in distilled water, followed by purging the dispersion with argon for 2 h. Then, the sample was transferred to a Float-A-Lyzer dialysis tube (100 kDa MWCO) and dialyzed against the dialysis buffer (50 mM Tris, 0.05% NaN_3, 1 mM KF; pH 7.5) at 4 °C for 3 days (buffer exchanged 3 times a day). The purified UCNP-SA conjugate was stored at 4 °C.[3–5]

1.2 Characterization of UCNPs

UCNPs were characterized by transmission electron microscopy (TEM) and dynamic light scattering (DLS). For the TEM analysis, a dispersion of UCNPs in cyclohexane (5 µL) was dispensed on a copper grid coated with 12 nm continual carbon foil, and the grid was dried on air at laboratory temperature. TEM images were taken by Titan Themis (FEI, Czech Republic) with FEI Ceta 16-megapixel CMOS camera. The size of individual particles was analyzed in ImageJ software (National Institutes of Health, USA).[6]

DLS analysis of hydrodynamic diameters of UCNPs and their conjugates was carried out using Zetasizer Nano ZS (Malvern, United Kingdom). The particles were diluted to a final concentration of 20 µg/mL with cyclohexane (in the case of oleic acid-capped UCNPs) or dialysis buffer (in the case of alkyne-PEG-neridronate-UCNPs and UCNP-SA bioconjugates), and the measurement was carried out at 25°C.[3]

1.3 Biotinylation of polyclonal anti-HSA antibody

First, NHS-LC-biotin (Merck, Germany) was dissolved in dry DMF to obtain a concentration of 20 mg/mL. Then, 1.67 µL of the solution was added to 100 µL of the polyclonal anti-HSA antibody (10.9 mg/mL in PB), followed by shaking for 10 min at RT and another addition of 1.67 µL of NHS-LC-biotin solution. The mixture was incubated for 2 h at RT with shaking and then overnight at 4 °C. The biotinylated antibody was purified by centrifuge filtration using Amicon Ultra (100 kDa MWCO), diluted in PB to a final concentration of 2 mg/mL, and stored at 4 °C.[2]

1.4 QD-based dot-blot immunoassay for HSA

Streptavidin-conjugated Cd-based core/shell quantum dots (QD-SA) were employed as a reference label in the dot-blot immunoassay for HSA. The same assay protocol as for the UCNP-based labels was utilized, with all the optimized parameters (coating time of 15 min, AL-01 concentration of 100 µg/mL, blocking with a mixture of 20% SB and 20% PFB in WB, biotinylated anti-HSA antibody concentration of 0.5 µg/mL, incubation times of 15 min, and AS3 assay buffer). The only difference was the last labeling step, where 1.9 mL of QD-SA dispersion (1 or 5 nM) was used per strip, followed by the incubation for 1 h. After the last washing step, the strips were left to dry in the dark and scanned using the UPCON S-Pro reader. A xenon flash lamp with a 340/70 nm excitation filter was used for the excitation of QDs, and the emission was collected through a 605/55 nm emission filter utilizing a D400 dichroic mirror.

1.5 Upconversion-linked immunosorbent assays

The polystyrene 96-well high-binding microtiter plate (MTP) with a clear bottom (Microlon; Greiner Bio-One, Austria) was coated with the monoclonal anti-HSA antibody AL-01 (1 µg/mL in CB, 100 µL/well) and incubated for 18 h at 4 °C. Then, the MTP was washed 4 times using a HydroFlex washer (Tecan, Switzerland); each washing step consisted of adding 250 µL of WB to each well with subsequent aspiration. The same protocol was used for all the washing steps during the ULISA procedure. Afterward, the MTP was blocked with a blocking buffer (20% SuperBlock in WB) for 1 h at RT with slow shaking. Then, the MTP was washed, and serial dilutions of HSA (from 10^{-3} to 10^{3} ng/mL) in assay buffer (AS1, containing 10% SuperBlock) or in 25% urine in AS1 were added (100 µL/well); each concentration was prepared in triplicates. The MTP was incubated for 1 h at RT with slow shaking. After washing the MTP, 0.5 µg/mL of biotinylated polyclonal anti-HSA antibody A0433 in AS1 (100 µL/well) was added for 1 h at RT with slow shaking. Afterward, the MTP was washed and incubated with UCNP-SA conjugate in AS1 (3.25 µg/mL; 100 µl/well) for 1 h at RT with slow shaking. After the last washing, the MTP was left to dry at RT. The upconversion luminescence was then measured by the UPCON S-Pro reader equipped with a 980-nm excitation laser source, a 976/30 nm optical filter was used for excitation, and the emission of UCNPs was measured using a 540/60 nm filter utilizing a D800 dichroic mirror (950–1000 nm excitation, 500–720 nm emission). A raster of 8×8 points was measured in each well using a 1 mm step and 1 s integration time. The truncated average of upconversion luminescence intensity in each well was calculated by excluding the 8 highest and 8 lowest intensity values.

ULISA detection of PSA and cTn shared the same protocol with HSA detection, except for the used biomolecules. For PSA detection, monoclonal antibody ab403 (1 µg/mL) was coated to the MTP, standard dilutions of PSA (from 10^{-4} to 10^{2} ng/mL) in AS1 or 50% fetal bovine serum in AS1 were used as an analyte, and biotinylated polyclonal antibody BAF1344 (0.25 µg/mL) was used as the detection antibody. For cTn detection, a 1:1 mixture of MF4c and 19C7cc antibodies was used for coating, with the final concentration of each antibody in the mixture of 1 µg/mL. The cTn dilutions (from 10^{-3} to 10^{3} ng/mL) in AS1 or 25% fetal bovine serum in AS1 were used as an analyte. Biotinylated anti-cTn antibody 560cc (0.5 µg/mL) was used as the detection antibody.

2 Results and discussion

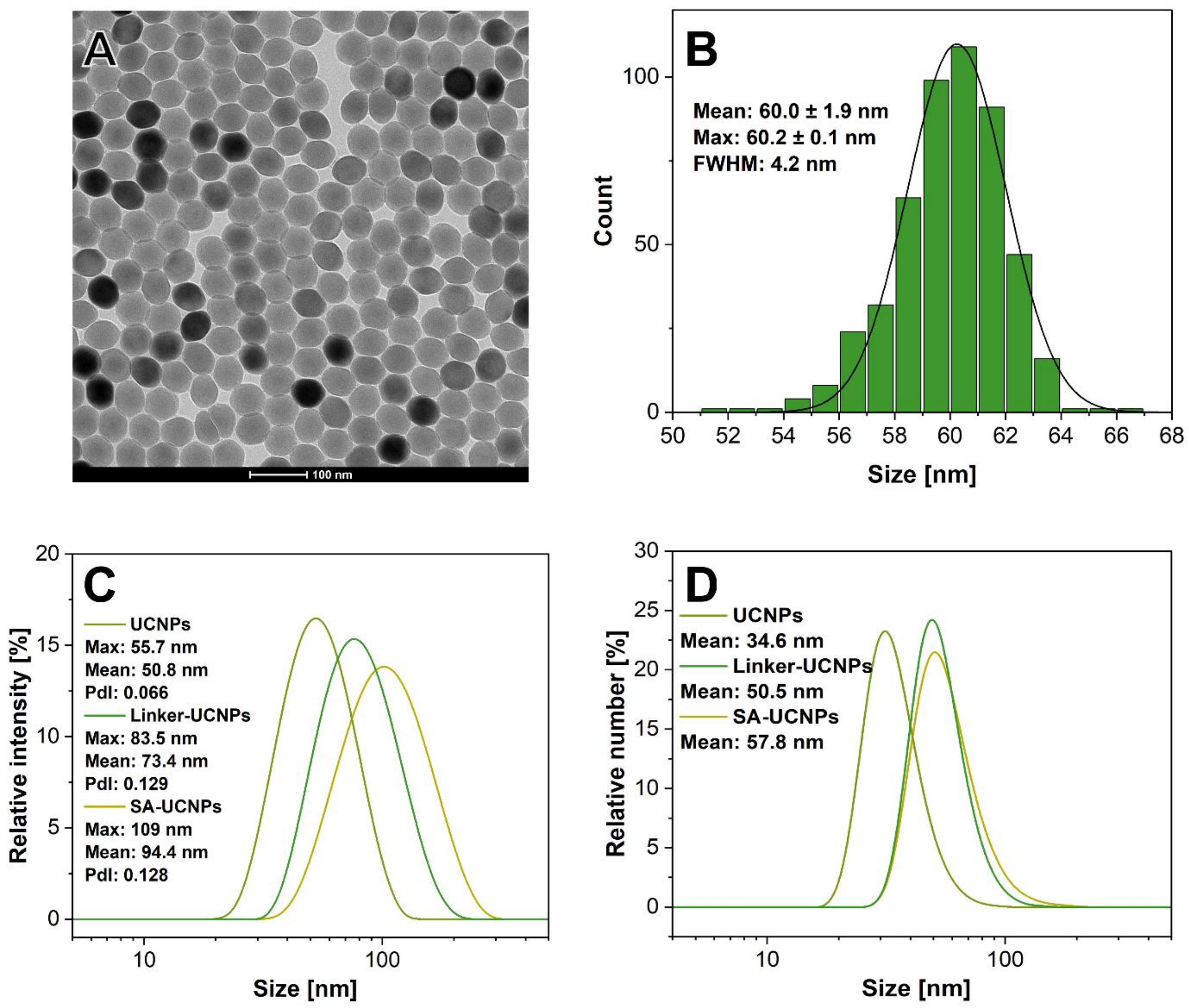

Figure S1: Characterization of UCNPs. **(A)** TEM image of oleic acid-capped UCNPs and **(B)** histogram of particle sizes with Gaussian fit. **(C)** Intensity and **(D)** number DLS particle size distribution of oleic acid-capped UCNPs, alkyne-PEG-neridronate-UCNPs (Linker-UCNPs), and UCNP-SA bioconjugates. B-spline function was used to connect the DLS data. Max represents the *x*-value of the peak maximum. Mean represents the average of the longest diameter of the UCNPs in the TEM or the average hydrodynamic diameter of the UCNPs in the DLS.

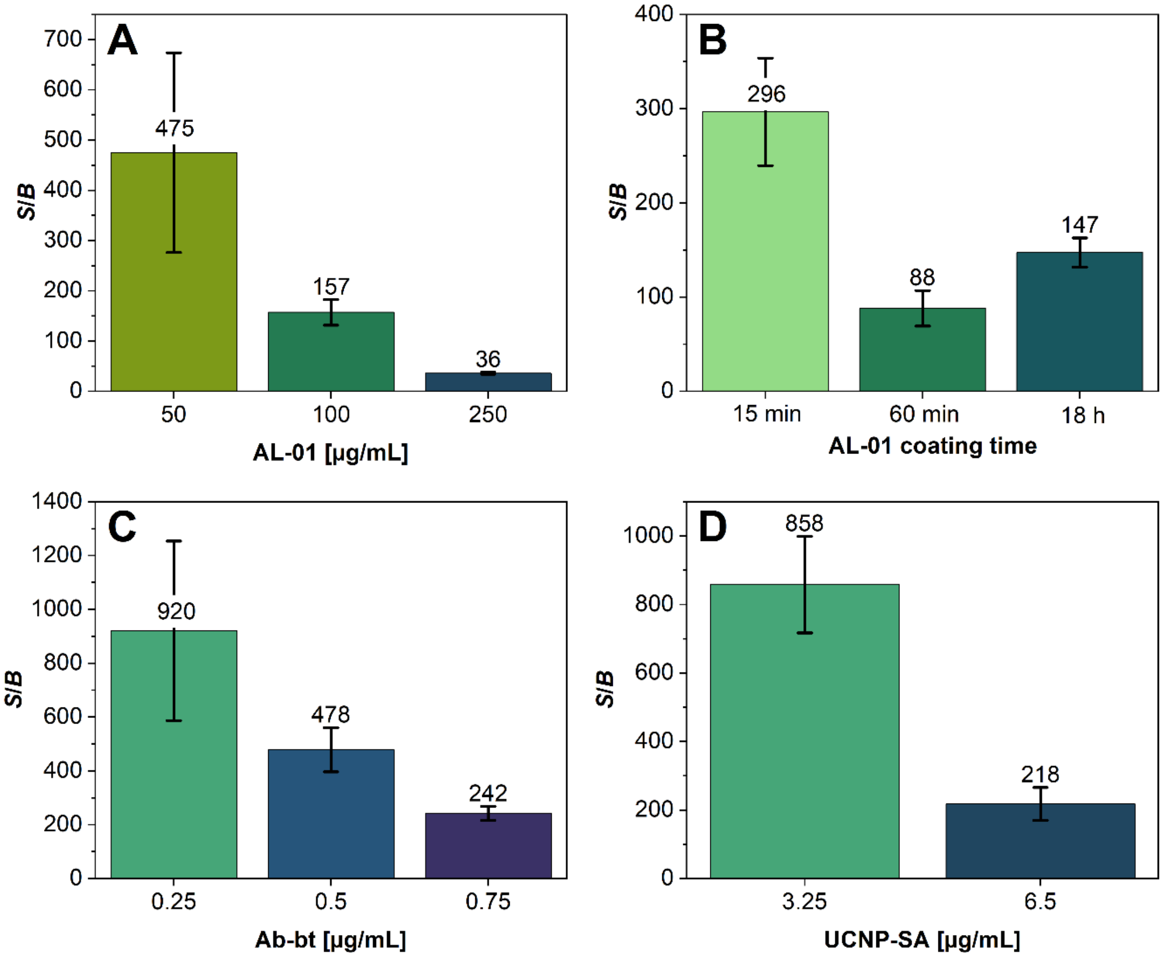

Figure S2: Optimizations of UCNP-based dot-blot for HSA. Dependence of *S*/*B* ratios on **(A)** the concentration of AL-01 capture antibody, **(B)** duration of coating of the AL-01 capture antibody, **(C)** concentration of the biotinylated detection antibody, and **(D)** concentration of the UCNP-SA label. Error bars represent standard deviations.

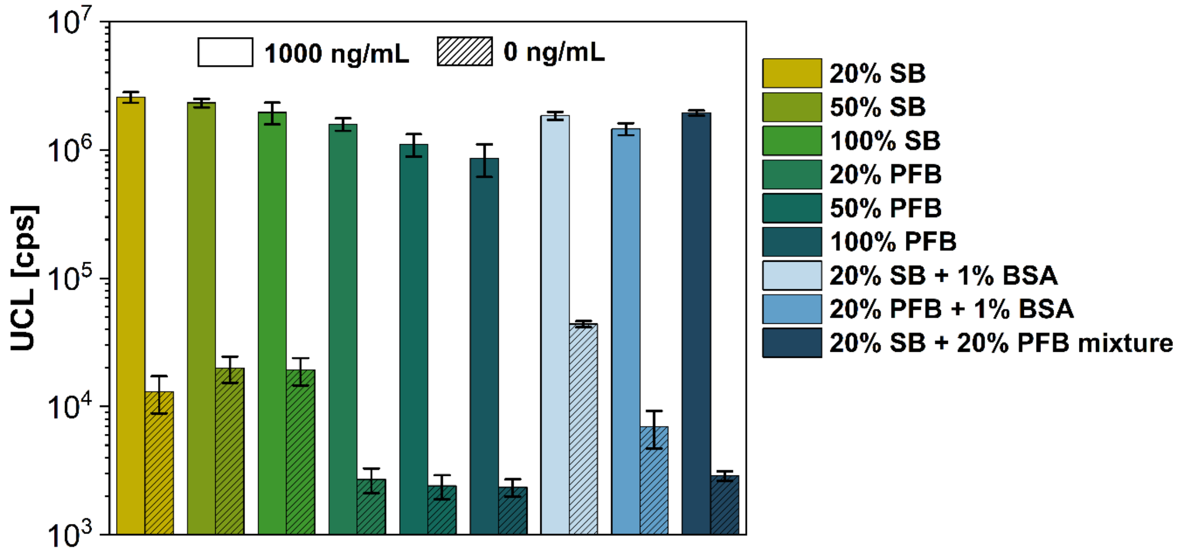

Figure S3: Intensities of HSA samples and blanks obtained using different blocking conditions. Error bars represent standard deviations.

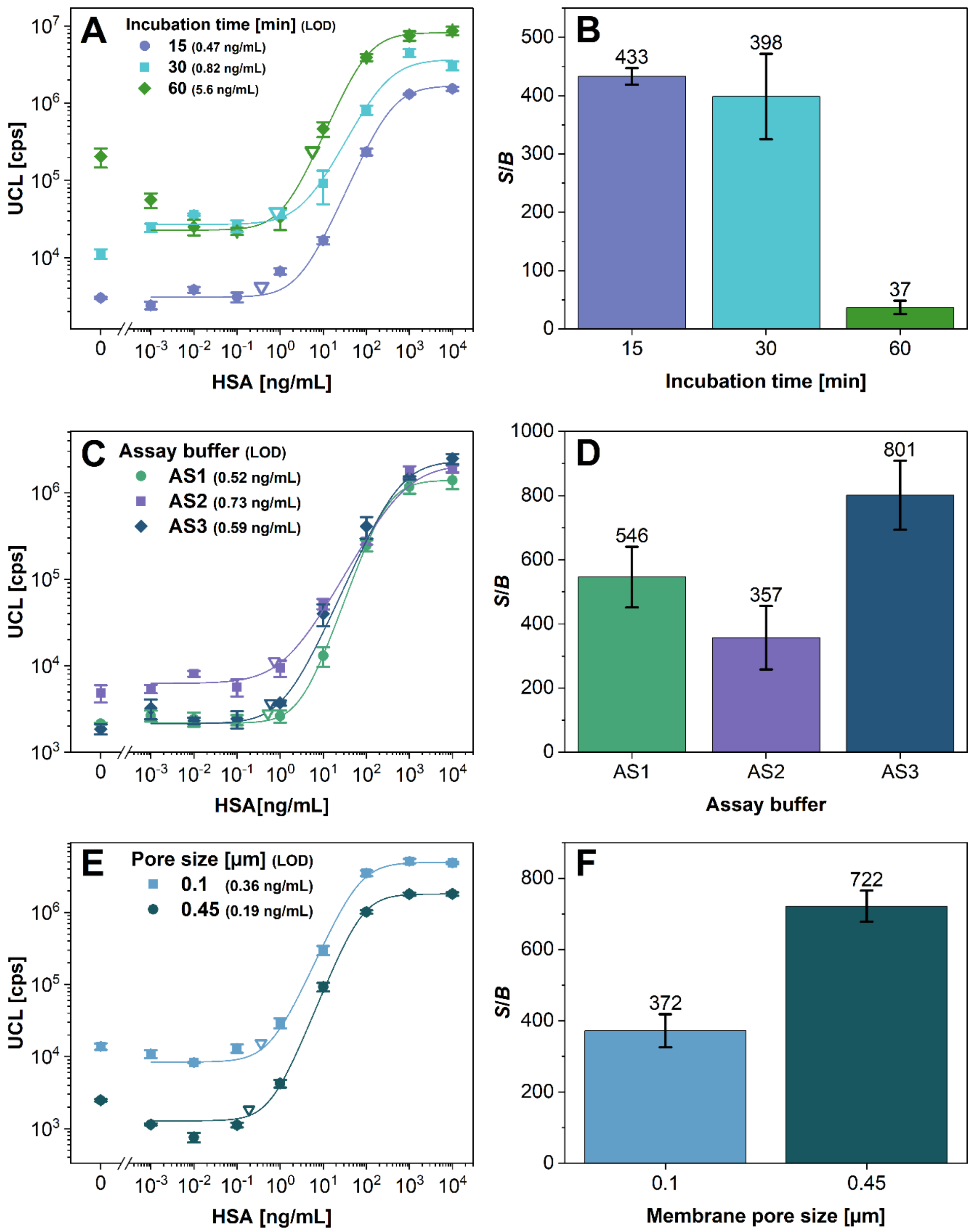

Figure S4: Optimization of incubation time, assay buffer, and membrane pore size in UCNP-based dot-blot for HSA. **(A)** Calibration curves with different times of incubation with biomolecules and UCNP-SA labels and **(B)** corresponding *S*/*B* ratios. **(C)** Calibration curves for different assay buffer compositions and **(D)** corresponding *S*/*B* ratios. **(E)** Calibration curves for different nitrocellulose membrane pore sizes and **(F)** corresponding *S*/*B* ratios. Error bars represent standard deviations, and empty triangles indicate the LODs.

Table S1: Summary of the optimized UCNP-based dot-blot assay parameters.

**Parameter**	**Optimized value**
Coating time of capture Ab	15 min
Incubation times (biomolecules and UCNP-SA)	15 min
Blocking	20% SB + 20% PFB mixture in WB
Blocking agent in assay buffer	10% PFB
Capture Ab concentration	100 µg/mL
Biotinylated detection Ab concentration	0.5 µg/mL
UCNP-SA concentration	6.5 µg/mL
Membrane pore size	0.45 µm

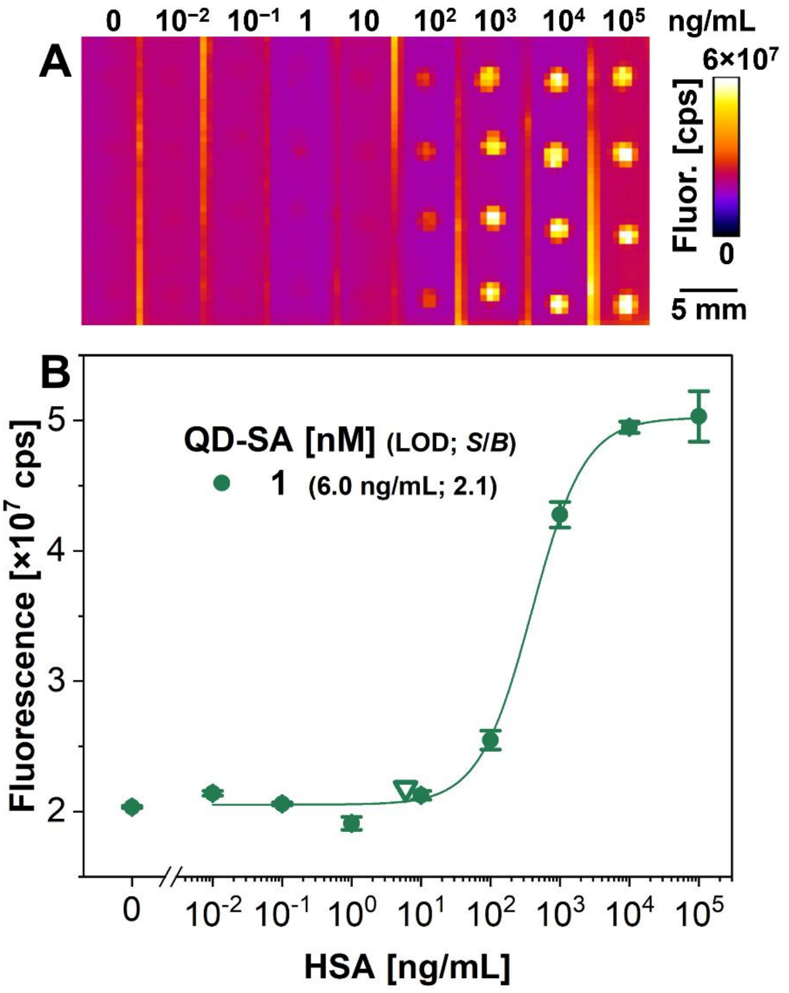

Figure S5: Dot-blot immunoassay for HSA detection with QD-SA labels. **(A)** Fluorescence intensity scan in pseudocolor scale of immunoassay with QD-SA concentration of 1 nM, and **(B)** the corresponding calibration curve. The HSA concentrations are indicated above the scan. The intensity scale in panel A was chosen to facilitate the visibility of lower signals; the graph in panel B was evaluated based on raw signal values. Error bars represent standard deviations, and empty triangle indicates the LOD.

Table S2: Autofluorescence background intensities of the MTP and nitrocellulose.

	**MTP**	**Nitrocellulose**	**Fold increase**
**Fluorescence [×10^7 cps]**	0.26 ± 0.07	2.04 ± 0.03	8.0×
**Upconversion [cps]**	22.3 ± 1.4	26.5 ± 0.6	1.2×

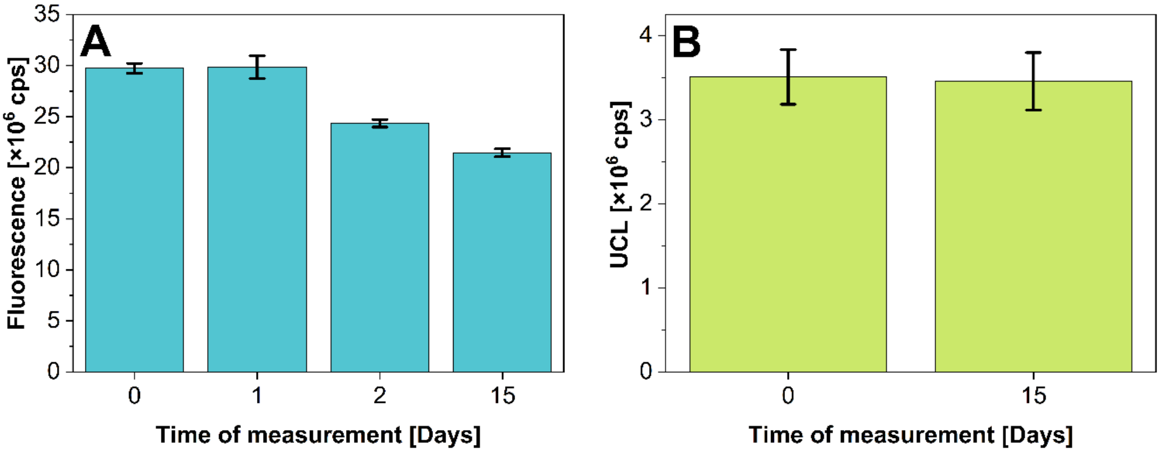

Figure S6: Comparison of the photostability of **(A)** QD-SA and **(B)** UCNP-SA labels.

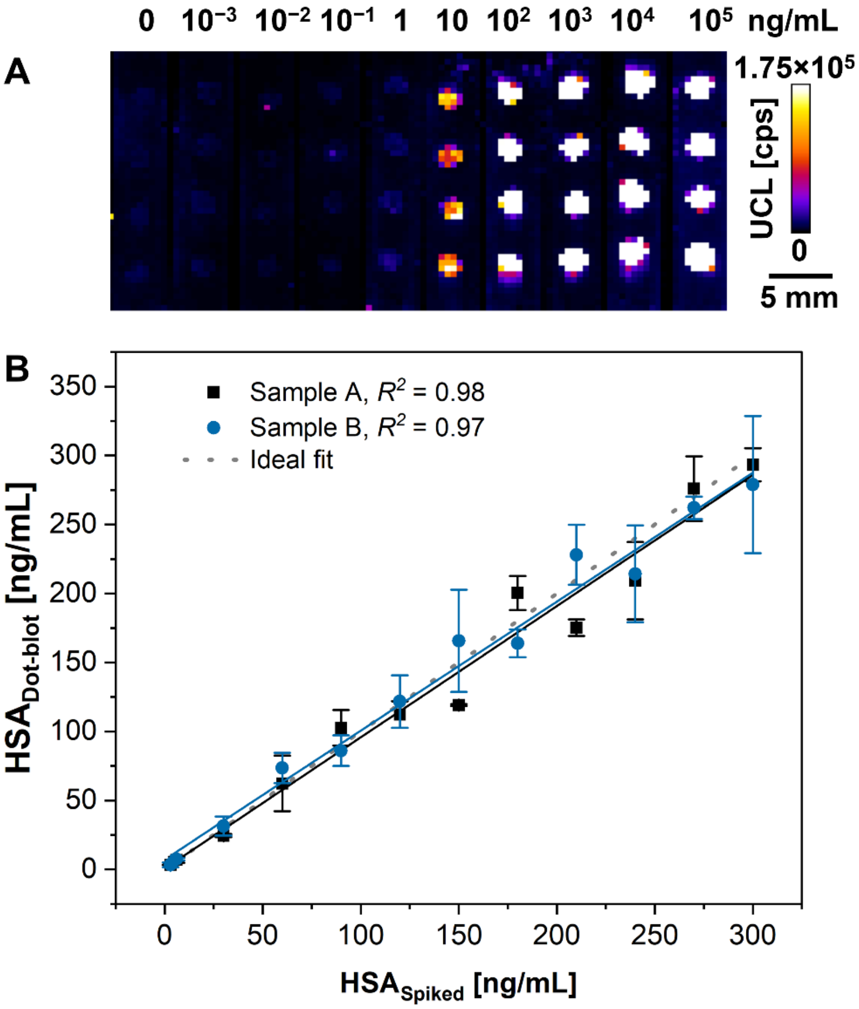

Figure S7: Detection of HSA in real samples. **(A)** Intensity scan in pseudocolor scale of the UCNP-based dot-blot for the detection of HSA in 25% urine. The HSA concentrations are indicated above the scan. The intensity scale was chosen to facilitate the visibility of lower signals. **(B)** Correlation between the concentrations of HSA spiked in the urine and the results obtained from the UCNP-based dot-blot assay. Error bars represent standard deviations.

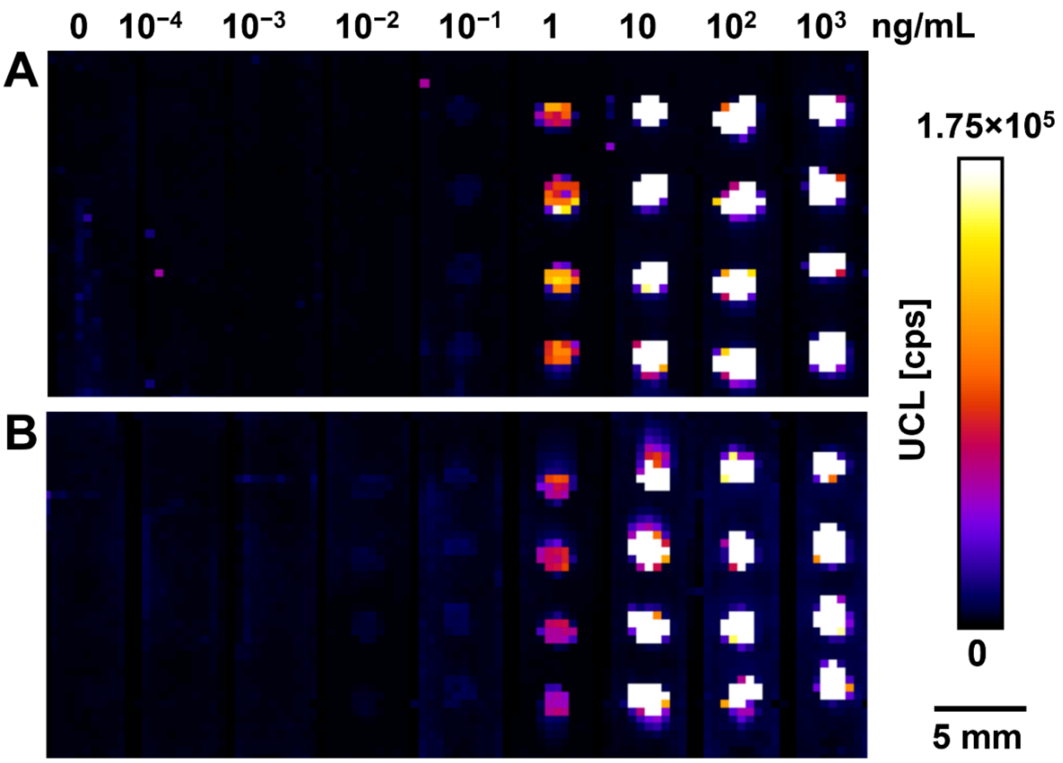

Figure S8: Intensity scans of the UCNP-based dot-blot for the detection of PSA in **(A)** assay buffer and **(B)** 50% serum. The PSA concentrations are indicated above the scans. The intensity pseudocolor scale was chosen to facilitate the visibility of lower signals.

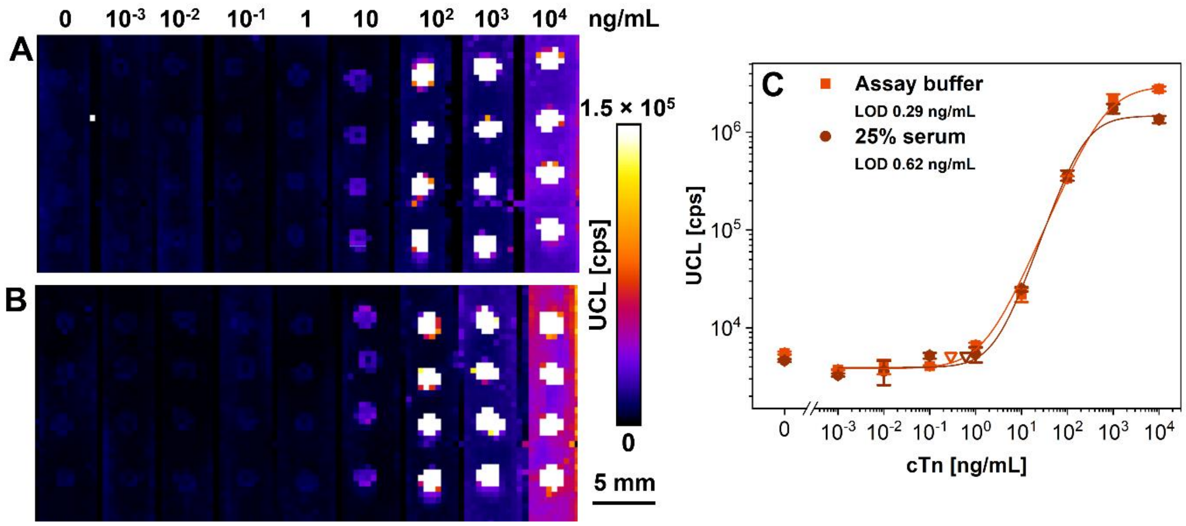

Figure S9: Intensity scans in pseudocolor scale of the UCNP-based dot-blot for the detection of cTn in **(A)** assay buffer and **(B)** 25% serum, and **(C)** the corresponding calibration curves. The cTn concentrations are indicated above the scans. The intensity scales in panels A and B were chosen to facilitate the visibility of lower signals; the graph in panel C was evaluated based on raw signal values. Error bars represent standard deviations, and empty triangles indicate the LODs.

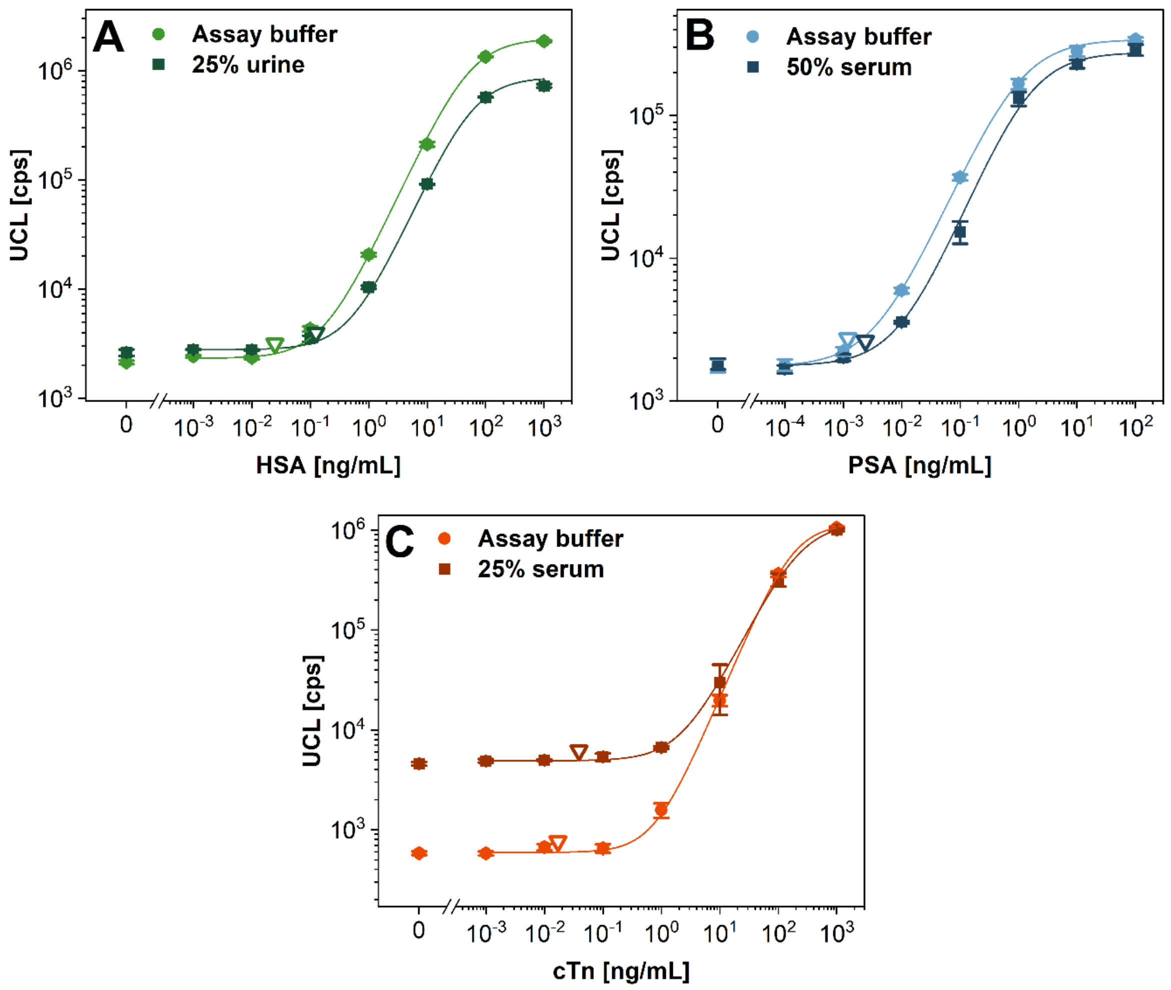

Figure S10: Calibration curves of MTP-based ULISA for the detection of **(A)** HSA, **(B)** PSA, and **(C)** cTn. Error bars represent standard deviations, and empty triangles indicate the LODs.

3 References

(1) Hlaváček, A.; Farka, Z.; Mickert, M. J.; Kostiv, U.; Brandmeier, J. C.; Horák, D.; Skládal, P.; Foret, F.; Gorris, H. H. Bioconjugates of Photon-Upconversion Nanoparticles for Cancer Biomarker Detection and Imaging. *Nat Protoc* **2022**, *17* (4), 1028–1072. https://doi.org/10.1038/s41596-021-00670-7.

(2) Brandmeier, J. C.; Jurga, N.; Grzyb, T.; Hlaváček, A.; Obořilová, R.; Skládal, P.; Farka, Z.; Gorris, H. H. Digital and Analog Detection of SARS-CoV-2 Nucleocapsid Protein via an Upconversion-Linked Immunosorbent Assay. *Anal Chem* **2023**, *95* (10), 4753–4759. https://doi.org/10.1021/acs.analchem.2c05670.

(3) Makhneva, E.; Sklenárová, D.; Brandmeier, J. C.; Hlaváček, A.; Gorris, H. H.; Skládal, P.; Farka, Z. Influence of Label and Solid Support on the Performance of Heterogeneous Immunoassays. *Anal Chem* **2022**, *94* (47), 16376–16383. https://doi.org/10.1021/acs.analchem.2c03543.

(4) Brandmeier, J. C.; Raiko, K.; Farka, Z.; Peltomaa, R.; Mickert, M. J.; Hlaváček, A.; Skládal, P.; Soukka, T.; Gorris, H. H. Effect of Particle Size and Surface Chemistry of Photon-Upconversion Nanoparticles on Analog and Digital Immunoassays for Cardiac Troponin. *Adv Healthc Mater* **2021**, *10* (18). https://doi.org/10.1002/adhm.202100506.

(5) Pořízka, P.; Vytisková, K.; Obořilová, R.; Pastucha, M.; Gábriš, I.; Brandmeier, J. C.; Modlitbová, P.; Gorris, H. H.; Novotný, K.; Skládal, P.; Kaiser, J.; Farka, Z. Laser-Induced Breakdown Spectroscopy as a Readout Method for Immunocytochemistry with Upconversion Nanoparticles. *Microchim Acta* **2021**, *188* (5), 147. https://doi.org/10.1007/s00604-021-04816-y.

(6) Rueden, C. T.; Schindelin, J.; Hiner, M. C.; DeZonia, B. E.; Walter, A. E.; Arena, E. T.; Eliceiri, K. W. ImageJ2: ImageJ for the next Generation of Scientific Image Data. *BMC Bioinformatics* **2017**, *18* (1), 529. https://doi.org/10.1186/s12859-017-1934-z.
